# Priming with inflammatory cytokines is not a prerequisite to increase immune-suppressive effects and responsiveness of equine amniotic mesenchymal stromal cells

**DOI:** 10.1186/s13287-020-01611-z

**Published:** 2020-03-04

**Authors:** Anna Lange-Consiglio, Pietro Romele, Marta Magatti, Antonietta Silini, Antonella Idda, Nicola Antonio Martino, Fausto Cremonesi, Ornella Parolini

**Affiliations:** 1 Department of Veterinary Medicine (DIMEVET), Università degli Studi di Milano, Via dell’Università 6, 26900 Lodi, Italy; 2 Centro Ricerca E. Menni, Fondazione Poliambulanza di Brescia, Via Bissolati 57, 25124 Brescia, Italy; 3 Department of Veterinary Science, University of Torino, Via Leonardo da Vinci 44, 10095 Turin, Italy; 4 Department of Life Scince and Public Health, Università Cattolica del Sacro Cuore di Roma, Largo F. Vito 1, 00168 Rome, Italy

**Keywords:** Equine, Amniotic stromal-derived cells, PBMC proliferation, Priming, Pro-inflammatory cytokines

## Abstract

**Background:**

Equine amniotic mesenchymal stromal cells (AMSCs) and their conditioned *medium* (CM) were evaluated for their ability to inhibit in vitro proliferation of peripheral blood mononuclear cells (PBMCs) with and without priming. Additionally, AMSC immunogenicity was assessed by expression of MHCI and MHCII and their ability to counteract the in vitro inflammatory process.

**Methods:**

Horse PBMC proliferation was induced with phytohemagglutinin. AMSC priming was performed with 10 ng/ml of TNF-α, 100 ng/ml of IFN-γ, and a combination of 5 ng/ml of TNF-α and 50 ng/ml of IFN-γ.

The CM generated from naïve unprimed and primed AMSCs was also tested to evaluate its effects on equine endometrial cells in an in vitro inflammatory model induced by LPS. Immunogenicity marker expression (MHCI and II) was evaluated by qRT-PCR and by flow cytometry.

**Results:**

Priming does not increase MHCI and II expression. Furthermore, the inhibition of PBMC proliferation was comparable between naïve and conditioned cells, with the exception of AMSCs primed with both TNF-α and IFN-γ that had a reduced capacity to inhibit T cell proliferation. However, AMSC viability was lower after priming than under other experimental conditions. CM from naïve and primed AMSCs strongly inhibited PBMC proliferation and counteracted the inflammatory process, rescuing about 65% of endometrial cells treated by LPS.

**Conclusion:**

AMSCs and their CM have a strong capacity to inhibit PBMC proliferation, and priming is not necessary to improve their immunosuppressive activity or reactivity in an inflammatory in vitro model.

## Background

Mesenchymal stromal cells (MSCs) have important properties of self-renewal, high proliferation, and differentiation potential, as well as their immunomodulatory features. They are a heterogeneous cell population found in the stroma of various adult, fetal, and extra-fetal tissues where they act as progenitor cells during natural tissue turnover. Extra-fetal tissues such as the umbilical cord, amniotic fluid, amnion, and placenta do not come with the ethical issues related to embryonic cells nor require invasive procedures involved in bone marrow harvesting. In addition, MSCs share the general properties of MSCs and, in some cases, have more potent immunomodulatory properties than adult counterparts [1, 2]. Immunomodulation is a regulatory activity by which MSCs interact with the adaptive and innate immune cells [3], both in cell-to-cell contact and via paracrine signaling, with the potential either to drive the inflammatory response toward its resolution or to strengthen it, depending on the surrounding microenvironment [4]. This property was first described by Bartholomew et al. [5], who reported the ability of MSCs to suppress lymphocyte proliferation in vitro and prevent rejection of in vivo allograft.

According to the polarization paradigm, quiescent MSCs do not possess immunomodulatory properties [6] and naïve MSCs can be polarized by environmental stimuli to acquire a pro-inflammatory (MSC1) or an immunosuppressive (MSC2) phenotype [7]. This switch is activated via Toll-like receptor family (TLR) members, respectively, by TLR4 and TLR3, which act upon recognition of pathogen-associated molecular patterns [8]. However, the inflammatory microenvironment is rich in other molecules that can prompt MSC activation such as those released by immune cells after tissue degradation. The inflammatory cytokine milieu plays a pivotal role in the switch of the MSC phenotype by determining functional maturation [4, 9]. Thus, active MSCs release cytokines, chemokines, growth factors, and microvesicles that act in a feedback loop to control the inflammatory response [10]. The damaged tissue is characterized, in vivo, by the presence of inflammatory cytokines that prime MSCs, acting as a sub-lethal event that can trigger an adaptive response of MSCs to future injury or damage [11]. However, the damaged tissue is also an inhospitable environment that can lead to the premature death of MSCs used for therapy [12] and to an increase in their immunogenicity [13].

In this context, MSC activation in vitro, defined also as priming or licensing, seems to be required to improve the survival rate of transplanted cells and to induce, simultaneously, the development of an immunosuppressive activity. Indeed, several studies indicate that MSCs need to be licensed by inflammatory stimuli, such as tumor necrosis factor-α (TNF-α), interferon-γ (IFN-γ), and interleukin-1 α/β, to exert or improve their immunosuppressive actions [14]. Thus, in recent years, several studies have evaluated whether preconditioning bone marrow-derived MSCs cultured in vitro with IFN-γ and TNF-α could elicit a stronger immunosuppressive activity [4, 15–17]. IFN-γ induces a transient expression of indoleamine-2,3 dioxygenase (IDO) which inhibits T cell activation, proliferation, and functionality due to l-tryptophane depletion [9]. In addition, IFN-γ priming induces the production of immunomodulatory molecules such as prostaglandin-E2 (PGE2), hepatocyte growth factor (HGF), transforming growth factor β1 (TGF-β1), and C-C motif chemokine ligand 2 (CCL2). Preconditioning with TNF-α has also been shown to enhance the production and release of these molecules, although to a lesser extent than IFN-γ priming. Bone marrow MSCs primed with a combination of IFN-γ and TNF-α had an even stronger immunosuppressive phenotype compared to those primed with a single factor, although significant differences were observed in the inhibitory potential of MSCs based on donor source [16]. However, priming with IFN-γ amplifies the expression of *MHCI* and *MHCII* in equine bone marrow-derived cells [18] increasing their immunogenicity.

We have previously reported that naïve amniotic mesenchymal stromal cells (AMSCs) from horse term placentae inhibit the proliferation of peripheral blood mononuclear cells (PBMCs) in vitro in both cell–cell contact and in a transwell culture system [19] without priming.

The aim of this paper is to understand if priming equine AMSCs in vitro with inflammatory cytokines improves their in vitro capability to inhibit PBMC proliferation and, eventually, alters their immunogenicity (expression of MHCI and MHCII markers). To this aim, AMSCs were stimulated by TNF-α and IFN-γ, molecules known to be present in inflammatory environments [20]. Since MSCs act via paracrine signaling, the CM generated from naïve and from primed AMSCs was also tested on equine endometrial cells in an inflammatory in vitro model to evaluate if priming makes the secretome more responsive in its reparative effect.

## Materials and methods

### Study design

The first part of the study evaluated the effect of AMSC priming with pro-inflammatory cytokines (TNF-α, IFN-γ, and their combination) on the expression of immunogenicity markers as well as MHCI and MHCII. The second part investigated the effect of naïve and primed AMSC, and their CM, on lymphocyte proliferation. The third part of the study evaluated the in vitro effect of CM derived from naïve (CM-CTR) and from primed AMSCs on endometrial cells treated with lipopolysaccharide (LPS). The cell viability, the apoptotic index, and the bioenergetic/oxidative status, expressed as mitochondria activity and intracellular sources of reactive oxygen species (ROS) levels, were determined.

The study was performed on AMSCs obtained from three distinct amniotic membranes (donors).

### Materials

Equine term placentas (*n* _3) were obtained following spontaneous vaginal delivery. All procedures to collect allanto-amniotic membranes were conducted following the standard veterinary practice and in accordance with the 2010/63 European Union directive on animal protection and Italian Law (D.L. No. 116/1992). Written informed consent from the owners was also obtained to collect placentas at delivery. Equine blood collection was approved by the University of Milan Ethics Committee (Protocol Number 41/15), and informed owner consent was obtained.

Uteri samples were collected from horses slaughtered in a national slaughterhouse under legal regulation.

Chemicals were obtained from Sigma-Aldrich Chemical (Milan, Italy) unless otherwise specified. LPS was purchased by Sigma-Aldrich Chemical (*Escherichia coli* 0:111B4; L2630 catalog number). Equine recombinant IFN-γ and equine recombinant TNF-α were purchased by R&D System (MN, USA). Tissue culture plastic dishes were purchased from Euroclone (Milan, Italy).

### Amniotic membrane collection and cell isolation

Allanto-amniotic membranes were obtained at the term from normal pregnancies of three horses and were processed separately as described by Lange Consiglio et al. [21]. First, the amniotic membrane was separated from its juxtaposed allantois and cut into small pieces (about 9 cm^2^ each). The amnion fragments underwent an incubation step with 2.4 U/ml dispase (Becton Dickinson, Milan, Italy) in phosphate buffer solution (PBS) for 9 min at 38.5 °C. Before completing the enzymatic digestion, the fragments were kept in high-glucose Dulbecco’s modified Eagle’s *medium* (HG-DMEM; EuroClone, Milan, Italy), supplemented with 10% heat-inactivated fetal bovine serum (FBS) and 2 mM l-glutamine for 5–10 min at room temperature. Final fragment digestion was achieved with 0.93 mg/ml collagenase type I and 20 mg/ml DNAse (Roche, Mannheim, Germany) for approximately 3 h at 38.5 °C. The debris was removed using a 100-μm cell strainer, and mobilized cells were collected by centrifugation at 200×*g* for 10 min. The AMSCs were cultured in HG-DMEM supplemented with 10% FBS, penicillin (100 UI/ml)-streptomycin (100 mg/ml), 0.25 mg/ml amphotericin B, 2 mM l-glutamine, and 10 ng/ml epidermal growth factor until passage 3.

### Endometrial cell isolation

Fresh uteri were collected from three different mares at the slaughterhouse intended for human consumption and unrelated to our studies. Samples were obtained from healthy normal-cycling mares in diestrus (middle-late luteal phase). Only uteri from mares with an obvious corpus luteum on the ovary were used for endometrial fragment collection and ensuing cell culture.

Endometrial cells from diestrus mare uteri were obtained according to the protocol described by Donofrio et al. [22] and slightly modified for equine cells. Briefly, the endometrium was digested in sterile-filtered Hank’s buffered salt solution supplemented with 2 mg/ml collagenase II, 4 mg/ml bovine serum albumin, and 0.4 mg/ml DNase I for 90 min at 38.5 °C in a shaking bath. Cells were then filtered through a membrane with a pore size of 80 μm, centrifuged at 200×*g* for 10 min, and then washed twice in PBS. This protocol allowed isolation of the endometrial stromal portion. Endometrial cell culture was established in HG-DMEM supplemented with 10% FBS, penicillin (100 UI/ml), streptomycin (100 μg/ml), 0.25 μg/ml amphotericin B, and 2 mM l-glutamine.

### Characterization by reverse transcription-PCR analysis of amniotic and endometrial cells

Some mesenchymal (*CD29*, *CD44*, *CD105*, *CD166*) and hematopoietic (*CD34*) markers were evaluated by RT-PCR analysis on AMSCs at P3. Equine-specific oligonucleotide primers and conventional PCR were used for the standard characterization of these cells as reported by Lange-Consiglio et al. [21]. Specific endometrial genes, such as progesterone receptor (PR), membrane-associated progesterone receptor (MPR), membrane-bound intracellular progesterone receptor component 1 (PGRMC1), and homeobox protein Hox-A9-like (HOXA-9), were analyzed on endometrial cells immediately after their isolation as reported by Corradetti et al. [23]. *GAPDH* was employed as reference gene.

### Priming with pro-inflammatory cytokines

Equine AMSCs were seeded at a density of 3 × 10^3^ on 12-well plates and stimulated for 96 h at 38.5 °C in 5% of CO_2_ with 10 ng/ml of TNF-α, 100 ng/ml of IFN-γ, as described for priming of equine BM-MSCs [18], and a combination of 5 ng/ml of TNF-α and 50 ng/ml of IFN-γ.

### Conditioned *medium* production and collection

Conditioned *medium* (CM) from naïve (control, CM-AMSC) and primed cells was obtained by culturing AMSCs at passage 3 in ultra-culture serum-free *medium* (Lonza, Basel, CH), without antibiotics for 96 h without replacement of *medium*.

Supernatants from each condition (CM-AMSC, CM-AMSC-TNF-α, CM-AMSC-IFN-γ, CM-AMSC-TNF-α+IFN-γ) were collected, centrifuged at 700×*g* to remove cellular debris and stored at − 80 °C. No further centrifugations or filtrations were performed on CM to preserve its total composition in terms of soluble (growth factors, chemokines, and other molecules) and non-soluble factors such as extra-cellular vesicles. This procedure was performed for cells obtained from three different placentas.

The collected supernatants were lyophilized and stored at 4 °C until use, at which point they were diluted in sterile water to one quarter of the initial volume.

After collection of CM, adherent cells were detached with 0.05% trypsin-EDTA (ethylenediaminetetraacetic acid) and frozen in HG-DMEM supplemented with 90% FBS and 10% DMSO (dimethyl sulfoxide) in liquid nitrogen until gene expression analysis and lymphocyte proliferation tests were carried out.

### Gene expression of immunogenicity markers

The mRNA expression levels of *MHCI* and *MHCII* were measured in three biological replicates. According to the manufacturer protocol, total RNA was isolated using the mirVana™ miRNA isolation Kit (Life Technologies) and stored at − 20 °C. The concentration and purity of RNAs were evaluated three times by the NanoQuant spectrophotometer (Thermo Scientific, USA). Eight samples, randomly chosen, were analyzed on Bioanalyzer 2100 using the Agilent RNA 6000 Pico Kit (Agilent) to verify the integrity of extracted RNA. According to the RNA quantity, each sample was normalized to the final RNA concentration of 10 ng/ul.

RT-PCRs were performed with the high capacity cDNA Reverse Trascription Kit (Applied Biosystems/Life Technologies, Carlsbad, CA, USA) using 100 ng of RNA per reaction.

The qPCR experiments were run in triplicates (technical replicates) using the qPCR protocol described by TaqMan Fast Gene Expression Assays (Life Tecnologies™) on 7500 Fast Real-time PCR System instrument (Applied Biosystems by Life Technologies™). Each target gene was amplified to assess gene expression. The glyceraldehyde 3-phosphate dehydrogenase gene (*GAPDH*) was the housekeeping control gene. The open-source PerlPrimer software v.1.1.17 was used to design equine-specific oligonucleotide primers based on available NCBI *Equus caballus* sequences or on mammal multi-aligned sequences. Primers were designed across an exon-exon junction in order to avoid DNA amplification (Table 1). The average target gene threshold cycle (ΔCt_g_) for each sample (calculated using the CT values of the technical replicates within each experimental condition) was normalized to the average GAPDH values (ΔCt_GAPDH_) of the same cDNA sample. Then, the expression variations calculated were normalized to internal control (i.e., CTR cell 3 h) using the ΔΔCt method. Finally, the fold change expression of each gene was calculated as 2^− ΔΔCT^ [24].

**Table 1:** Oligonucleotide sequences used for RT-PCR analysis

Markers	Sequences (5′➔3′)	Product size (bp)	Annealing temperature (°C)
Glyceraldehyde-3-phosphate dehydrogenase (*GAPDH*)	S: AGATCAAGAAGGTGGTGAAG A: TTGTCATACCAGGAAATGAGC	168	60
Major histocompatibility complex I (*MHC-I*)	S: GGAGAGGAGCAGAGATACA A: CTGTCACTGTTTGCAGTCT	218	55
Major histocompatibility complex II (*MHC-II*)	S: TCTACACCTGCCAAGTG A: CCACCATGCCCTTTCTG	178	55

### AMSC surface expression of MHC I and MHC II by flow cytometry

Aliquots of naïve and pre-conditioned AMSCs were analyzed at passage P3 with 100 μl Mouse anti Equine MHC Class 1 primary antibody (Bio-Rad, CA, USA; clone CVS22) and Mouse anti Equine MHC Class II antibody (Bio-Rad; clone CVS20) in PBS 3% BSA for 45 min at room temperature in the dark. After incubation, MSC analyzed with MHC I and MHC II were washed in cold PBS and incubated in 100-μl secondary FITC-conjugated goat anti-Mouse IgG antibody (Bio-Rad) for 20 min on ice in the dark. Finally, labeled cells were washed twice in ice-cold PBS and analyzed using Epics Coulter flow cytometer (Beckman Coulter-IL, Fullerton, CA, USA). A minimum of 10,000 cells were acquired for each sample and analyzed in the FL1 channel. All analyses were based on control cells incubated with isotype-specific IgGs to establish the background signal. Off-line analysis of the flow cytometry files was performed using Weasel software v.2.5.

### Lymphocyte proliferation test

Horse peripheral blood mononuclear cells (PBMC) were obtained from heparinized whole blood samples using density gradient centrifugation (Lymphoprep; Axis-Shield, Oslo, Norway) at 500 g without brakes for 20 min at room temperature. Informed consent was obtained from the owners for blood collection.

To study the effects of equine AMSCs on lymphocyte proliferation in a cell-to-cell contact setting, different numbers of AMSCs from each amniotic membrane were seeded per well (0.1 × 10^5^, 0.5 × 10^5^, 1 × 10^5^) in RPMI complete *medium* supplemented with 10% heat-inactivated FBS, 2 mM l-glutamine, and penicillin 10,000 U/ml, streptomycin 10 mg/ml (P/S) and left to adhere overnight. The next day, AMSCs were gamma-irradiated (3000 cGy) to ensure that the proliferation observed could be attributed solely to the proliferation of responder lymphocytes. Then, 2 × 10^5^ equine peripheral blood mononuclear cells (PBMCs) were added to each well in ultra-culture serum-free *medium* supplemented with 2 mM l-glutamine (Sigma) and penicillin 10,000 U/ml, streptomycin 10 mg/ml (P/S).

To study the paracrine effects of equine AMSCs on lymphocyte proliferation, 10 μL, 50 μL, or 100 μL of CM-AMSC, CM-AMSC-TNF-α, CM-AMSC-IFN-γ, and CM-AMSC-TNF-α+IFN-γ of each amniotic membrane were added to 2 × 10^5^ PBMCs in each well. A non-conditioned *medium* (non-CM) used as a control was generated in the same way as above, except that no cells were cultured in the plates. Another control was represented by PBMCs alone (unstimulated). For both studies with AMSCs and CM, PBMCs were stimulated with 2 μg/mL phytohemagglutinin (PHA).

All cultures were carried out in triplicate in flat-bottomed 96-well tissue culture plates (Corning, Milan, Italy) in a final volume of 200 μl.

Lymphocyte proliferation was assessed after 3 days of culture by adding 0.67 μCi of [3H]-thymidine (Perkin Elmer) per well for 16–18 h. Cells were then harvested with a Filtermate Harvester (PerkinElmer), and [3H]-thymidine incorporation was measured using a microplate scintillation and luminescence counter (Top Count NXT; PerkinElmer).

### In vitro effect of CM on endometrial cell viability and apoptosis

The dose-response curve of LPS on endometrial cells was studied showing that 10 ng/ml of LPS for 24 h was most effective in inducing cellular stress evaluated by apoptosis [25]. Sixty thousand cells were incubated with LPS 10 ng/ml for 24 h and with 10% of CM-AMSC or CM derived from AMSCs primed (CM-AMSC-TNF-α, CM-AMSC-IFN-γ, CM-AMSC-TNF-α+IFN-γ).

Endometrial cells alone, endometrial cells with LPS, and endometrial cells with different CM were used as controls.

Cell viability and apoptosis of endometrial cells stressed by LPS and treated with 10% of different CM were assessed using a combination of acridine orange (AO) and propidium iodide (PI) staining. Briefly, reagent solutions were prepared by dissolving 3 mg of PI in 1 ml of absolute ethanol and 5 mg of AO in 1 ml of ethanol and stored at 4 °C. A working solution was obtained by mixing 1 μl of AO solution, 1 μl of PI solution, and 1 ml phosphate buffer solution (PBS).

At each time point, the cell monolayer was trypsinized and the cell suspension was centrifuged at 250 g for 10 min. Fifty microliters of pellets were diluted with 50 μl of working solution. Examination under a fluorescence light microscope (Olympus BX51, Japan) at a magnification of ×40 was performed immediately. Acridine orange dye was excited at 460 nm while the emission wavelength was set at 650 nm. Propidium iodide was excited at 535 nm while the emission wavelength was set at 617 nm. According to the literature [26], cell staining and morphology were considered to define viable cells, apoptotic cells, and necrotic cells. Under a fluorescence microscope, viable cells showed normal nuclei staining with green chromatin; apoptotic cells showed condensed or fragmented chromatin (green or orange) and budding of the cell membrane; necrotic cells had similar normal nuclei staining as viable cells except the chromatin was orange or red instead of green.

### In vitro effect of CM on endometrial cell mitochondrial activity and ROS levels

For assessing the bioenergetic/oxidative status of endometrial cells stressed by LPS and treated with 10% CM, 1 × 10^4^ cells were seeded in 35 mm Petri dishes onto coverslips and cultured in the conditions described previously, until 50% confluence was reached. To investigate mitochondrial distribution and apparent energy status, cells were washed three times in PBS with 3% bovine serum albumin (BSA) and incubated for 30 min in the same medium containing 280 nM MitoTracker Orange CMTM Ros (Molecular Probes, OR, USA) at 38.5 °C under 5% CO_2_ [27, 28]. The probe contains a thiol-reactive chloromethyl moiety and passively enters the cell membrane. The probe is readily sequestered only by active mitochondria, and it can react with accessible thiol groups on peptides and proteins to form an aldehyde-fixable conjugate [29, 30].

After incubation with a mitochondrial probe, cells were washed three times in PBS with 0.3% BSA and incubated for 15 min in the same media containing 10 μM 2′,7′-dichlorodihydrofluorescein diacetate (H2DCF-DA) [31–33] to detect intracellular ROS. This probe is membrane-permeant and can diffuse into cells. Once inside the cell, the acetate groups are hydrolyzed by intracellular esterase producing 2′,7′-dichlorodihydrofluorescein (H2DCF) which is a polar molecule and thus retained inside the cell. H2DCF fluoresces when it is oxidized by H_2_O_2_ or lipid peroxides to produce 2′,7′-dichlorofluorescein (DCF). The level of DCF is related linearly to that of peroxides, and thus, its fluorescence provides a measure of peroxide levels [30]. The cells were then fixed with 2% paraformaldehyde in PBS for 2 h, stained with 2.5 mg/mL Hoechst 33258 in 3:1 of glycerol to PBS solution, and mounted onto slides in the same solution. Evaluation of fluorescence intensities and nuclear chromatin was performed using a Zeiss epifluorescence microscope at ×200 magnification. For the quantification analysis, 5 randomly selected microscope fields were evaluated (around 300 cells for each experimental condition) by using ImageJ software (Rasband, W.S., ImageJ, US National Institutes of Health, Bethesda, MD, USA, https://imagej.nih.gov/ij/, 1997–2018.). Fluorescence intensities are expressed as arbitrary densitometric units (ADU).

### Statistical analysis

Data are expressed as mean ± standard deviation. Differences between AMSC or CM-AMSC and controls were analyzed by the analysis of variance (ANOVA) followed by Tukey’s multiple comparisons test.

For immunogenicity markers, statistical differences were determined using ANOVA followed by Dunnett’s multiple comparison test, the Tukey–Kramer multiple comparisons test, or unpaired *t* test.

Differences were considered statistically significant if *P* was < 0.05.

Statistical analysis was performed with GraphPad Prism 6 Software (GraphPad Software, San Diego, CA, USA).

## Results

### Tissue collection and cell isolation

**Fig. 1:**
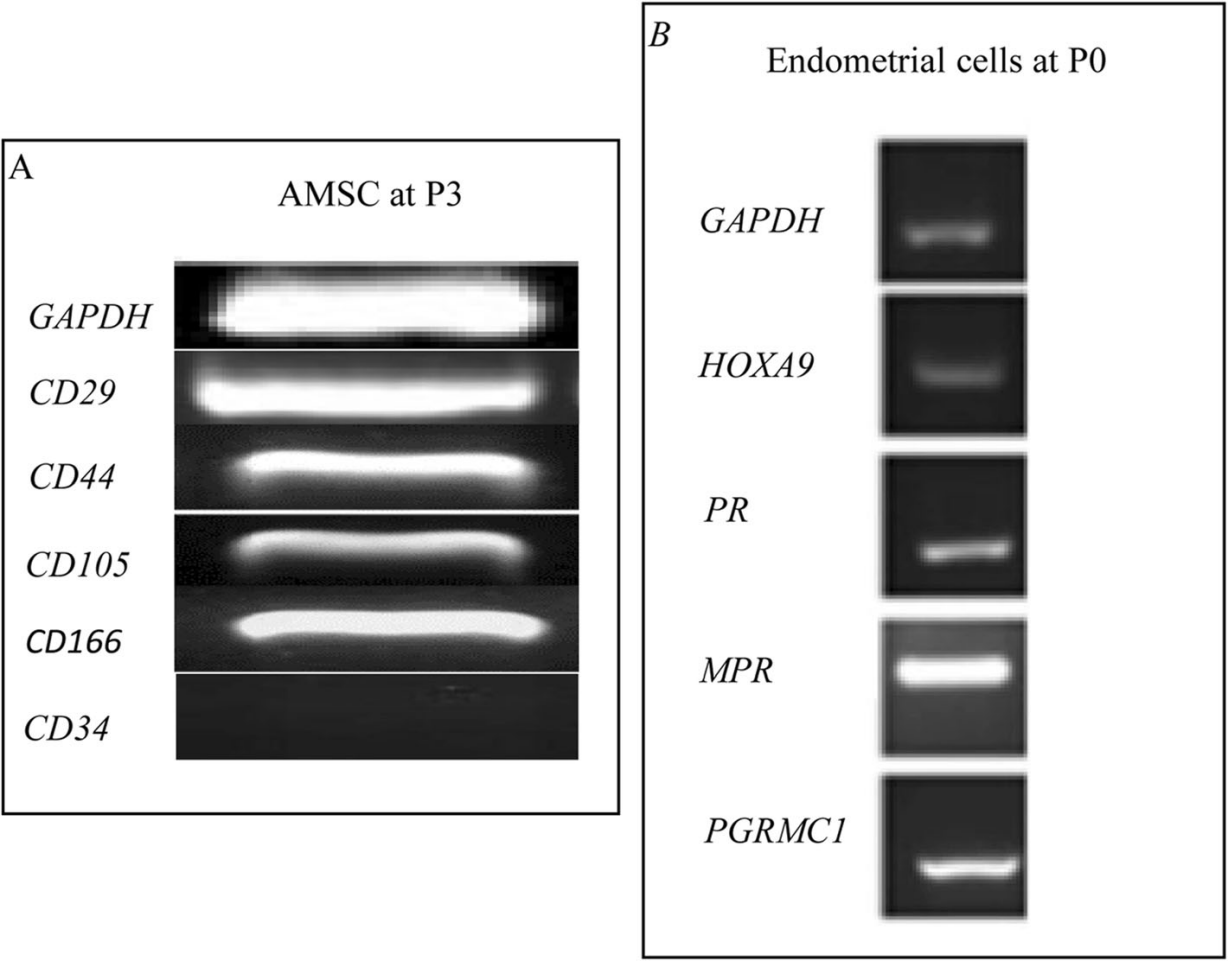
Molecular biology analyses. **a** Mesenchymal stromal cell phenotype of AMSCs at P3. **b** Molecular expressions of endometrial cells at P0

Cell selection was based on the ability of cells to adhere to plastic. Trypan blue exclusion assay showed that the initial viability was > 90% for AMSCs and > 85% for endometrial cells. As expected, for all mesenchymal stromal cell phenotypes, the AMSCs at P3 express CD29, CD44, CD106, CD105, and lack CD34 (Fig. 1a). Moreover, AMSCs are able to differentiate in mesenchymal (osteogenic, adipogenic, and chondrogenic) and ectodermic lineages (neurogenic) as reported by Lange-Consiglio et al. [21].

Molecular biology analyses of endometrial cells at P0 confirmed the expression of PR, MPR, PGRMC1, and HOXA-9 (Fig. 1b).

### Gene expression of immunogenicity markers

**Fig. 2:**
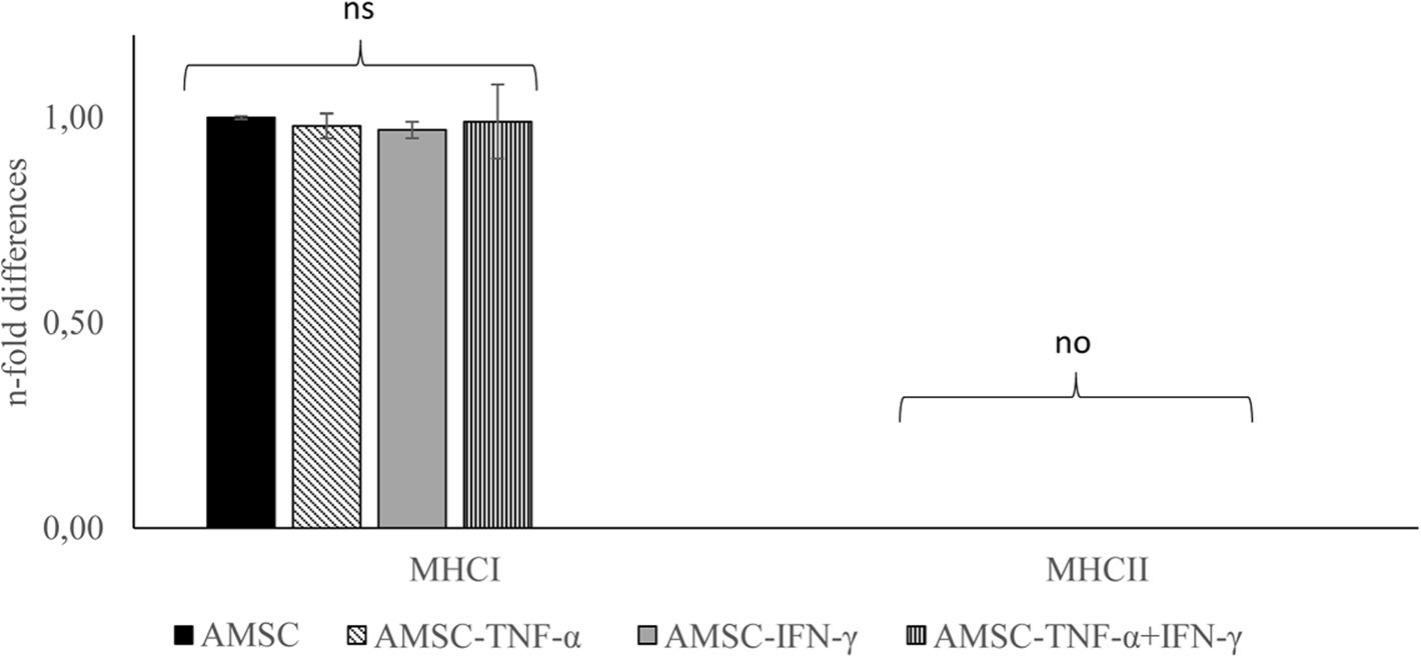
Equine amniotic-derived mesenchymal cell (AMSC) gene expression studied by real-time quantitative polymerase chain reaction (qRT-PCR) under the four culture conditions: AMSC, AMSC conditioning by TNF-α (AMSC-TNF-α), IFN-γ (AMSC-IFN-γ), or a combination of TNF-α and IFN-γ (AMSC-TNF-α+IFN-γ). Gene expression is shown as mean ± SD (*n* = 3) fold change versus standard condition (AMSC). ns = not significant; no = not observed

The gene expression of *MHCI* and *MHCII* after priming was assessed. In all tested conditions, the cells had a similar expression of MHC I with values similar to control cells and lacked the gene expression of MHC II (Fig. 2).

### AMSC surface expression of MHC I and MHC II

**Fig. 3:**
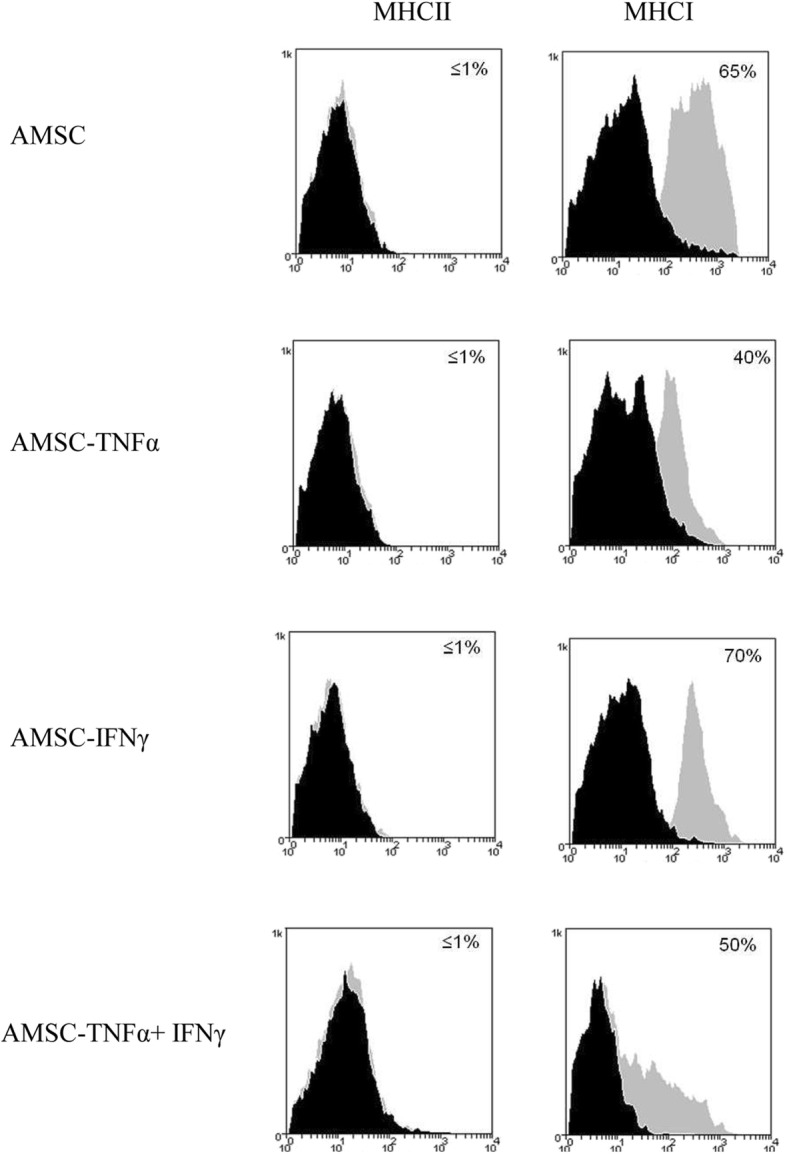
Flow cytometry analysis of MCHI and MCHII antigen. Histograms represent a relative number of cells vs fluorescence intensity (FL1). Black histograms indicate background fluorescence intensity of cells labeled with isotype control antibodies only; gray histograms show positivity to the studied antibodies

Representative immunophenotype patterns of naïve and pre-conditioned AMSCs are reported in Fig. 3. All populations were negative for MHCII and positive for MHCI (average 56.25%, range 40–70%).

### Lymphocyte proliferation test

**Fig. 4:**
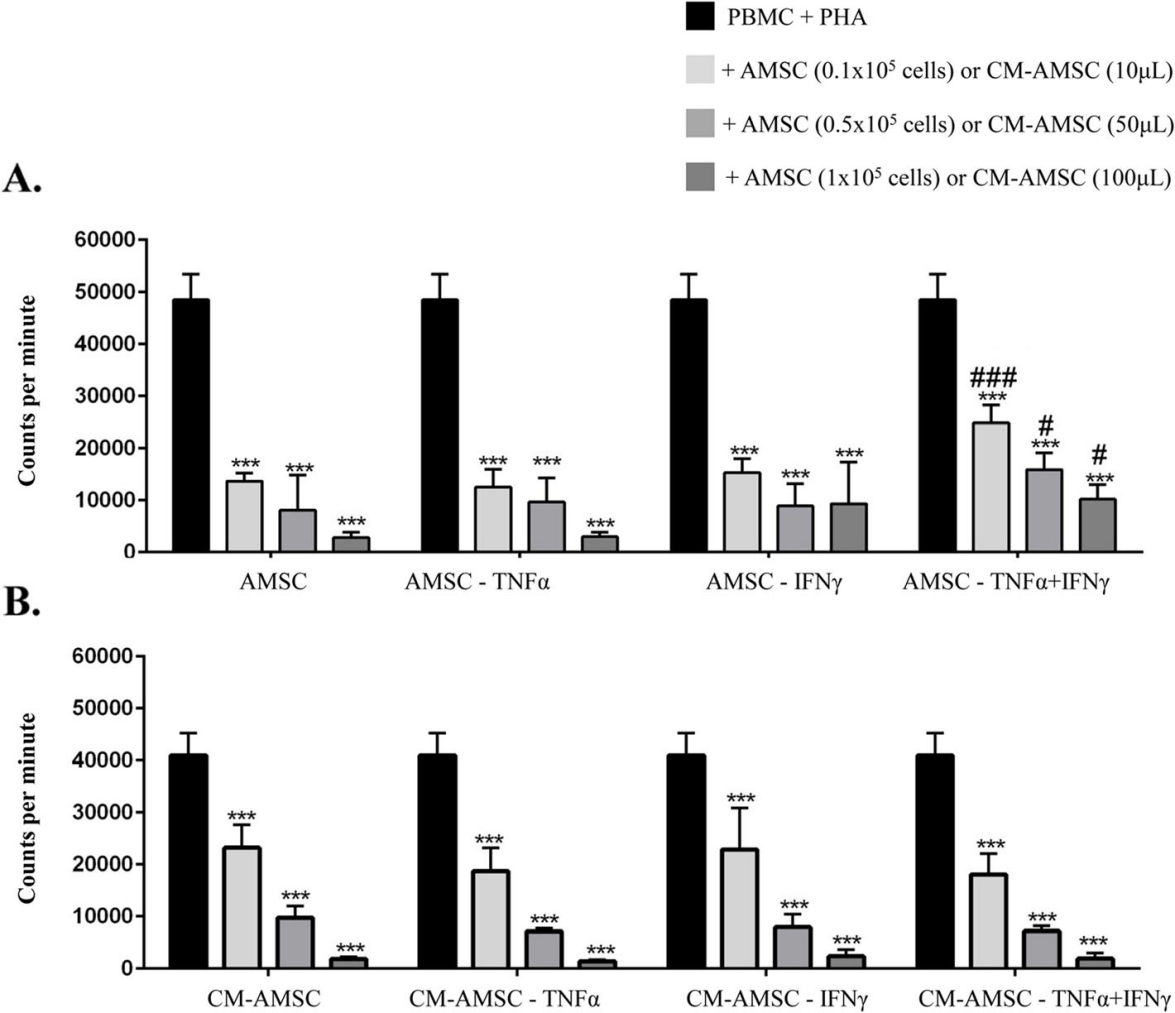
Effects of equine AMSC (**a**) and AMSC-derived conditioned medium (CM) (**b**) on the proliferation of PHA-stimulated equine lymphocytes. **a** Different experimental conditions represented are naïve AMSC, AMSC-TNF-α, AMSC-IFN-γ, and AMSC-TNF-α+IFN-γ. 1 × 10^5^, 0.5 × 10^5^, and 0.1 × 10^5^ AMSC/well were cultured in contact with 2 × 10^5^ PBMC stimulated with PHA. **b** Different experimental conditions represented are conditioned medium (CM) from naïve AMSC (CM-AMSC), CM from AMSC primed with TNF-α, with IFN-γ, or with both TNF-α+IFN-γ. Ten microliters, 50 μL, or 100 μL CM-AMSC/well was added to 2 × 10^5^ PBMC stimulated with PHA. Data representing the mean and standard deviation of three different AMSC preparations each of which was tested with two different lymphocyte donors, with the exception of the third AMSC which was tested with one lymphocyte donor. ^#^*p* < 0.05; *** and ^###^*p* < 0.001. * vs PBMC+PHA, # vs AMSC or CM-AMSC group

In all the conditions tested (naïve AMSC (CTR) and AMSC primed with TNF-α, or IFN-γ, or with a combination of TNF-α and IFN-γ), the proliferation of PBMC activated with PHA was inhibited in a dose-dependent manner (Fig. 4a) and stronger effects were observed at a PBMC: AMSC ratio of 1:0.5 and with 100 μL/well of CM. No significant differences were observed between naive AMSC and AMSC stimulated with TNF-α or IFN-γ (with 1 × 10^5^ AMSC the inhibition of PBMC proliferation was of 91.33 ± 5.03% for naïve AMSC, 85.00 ± 9.85% for TNF-α, 78.00 ± 17.52% for IFN-γ). Conversely, a combination of TNF-α and IFN-γ priming decreased capacity of AMSCs to inhibit proliferation when compared to naïve AMSCs (63.00 ± 26.96% inhibition; Fig. 4a). This difference was possibly due to the lower AMSC viability after double cytokine stimulation (58% ± 11.6%) when compared to the single cytokine stimulation (78% ± 2.7% for TNF-α and 77% ± 2.4% for IFN-γ) and to naïve AMSCs (72% ± 4.8%).

In addition, we showed that CM-AMSC can also inhibit the proliferation of PBMC in a dose-dependent manner (Fig. 4b). No differences were observed in the inhibitory ability between the different cytokine stimulation conditions: with 100 μL/well, 96.00 ± 1.00% inhibition with CM-AMSC, 97.00 ± 1.00% for CM-AMSC-TNF-α, 94.33 ± 2.89% for CM-AMSC-IFN-γ, and 95.67 ± 2.52 with the combination CM-AMSC-TNF-α+IFN-γ.

The same data are shown in Fig. 3 as count/minute.

### In vitro effect of CM on endometrial cell viability and apoptosis

Data for viability and apoptotic staining are reported in Table 2. These results demonstrate that LPS treatment induces 38.68% endometrial cell death and 39.66% apoptosis, with only 21.66% of live cells present at 24 h. In the presence of 10% CM-AMSC, the effect of LPS was countered, indeed, 65.59 ± 3.12% of endometrial cells were viable at 24 h of culture. In the presence of LPS and CM derived from priming with TNF-α, IFN-γ, and the combination of the 2 cytokines, endometrial cell viability was 64.31 ± 1.48%, 64.87 ± 5.24%, and 64.12 ± 0.95%, respectively, and there were no statistical significant differences compared to CM-AMSC.

**Table 2:** Rate of live, dead, and apoptotic cells in different experimental conditions

Condition	Live	Apoptotic	Dead
CTR	88.00 ± 2.12^a^	9.83 ± 1.23^a^	2.17 ± 1.02^a^
CM-AMSC	88.84 ± 1.28^a^	8.47 ± 1.94^a^	2.69 ± 0.82^a^
CM-AMSC-TNF-α	77.83 ± 0.77^b^	16.22 ± 0.74^b^	5.95 ± 0.03^b^
CM-AMSC-IFN-γ	77.09 ± 0.37^b^	15.84 ± 0.24^b^	7.07 ± 0.12^b^
CM-AMSC-TNF-α+IFN-γ	59.38 ± 0.59^c^	24.94 ± 1.86^c^	15.68 ± 1.27^c^
LPS	21.66 ± 6.88^d^	39.66 ± 9.81^d^	38.68 ± 6.69^d^
LPS+CM-AMSC	65.59 ± 2.55^e^	23.97 ± 2.59^e^	10.44 ± 2.20^e^
LPS+CM-AMSC-TNF-α	64.31 ± 1.48^e^	24.96 ± 2.73^e^	10.73 ± 1.30^e^
LPS+CM-AMSC-IFN-γ	64.87 ± 5.24^e^	24.00 ± 4.01^e^	11.13 ± 2.15^e^
LPS+ CM-AMSC-TNF-α+IFN-γ	64.12 ± 0.95^e^	22.46 ± 3.46^e^	13.42 ± 3.64^e^

Different small letters superscript (a, b) in the different columns indicate statistically different comparisons (*P* < 0.05)

### In vitro effect of CM on endometrial cell mitochondrial activity and ROS levels

**Fig. 5:**
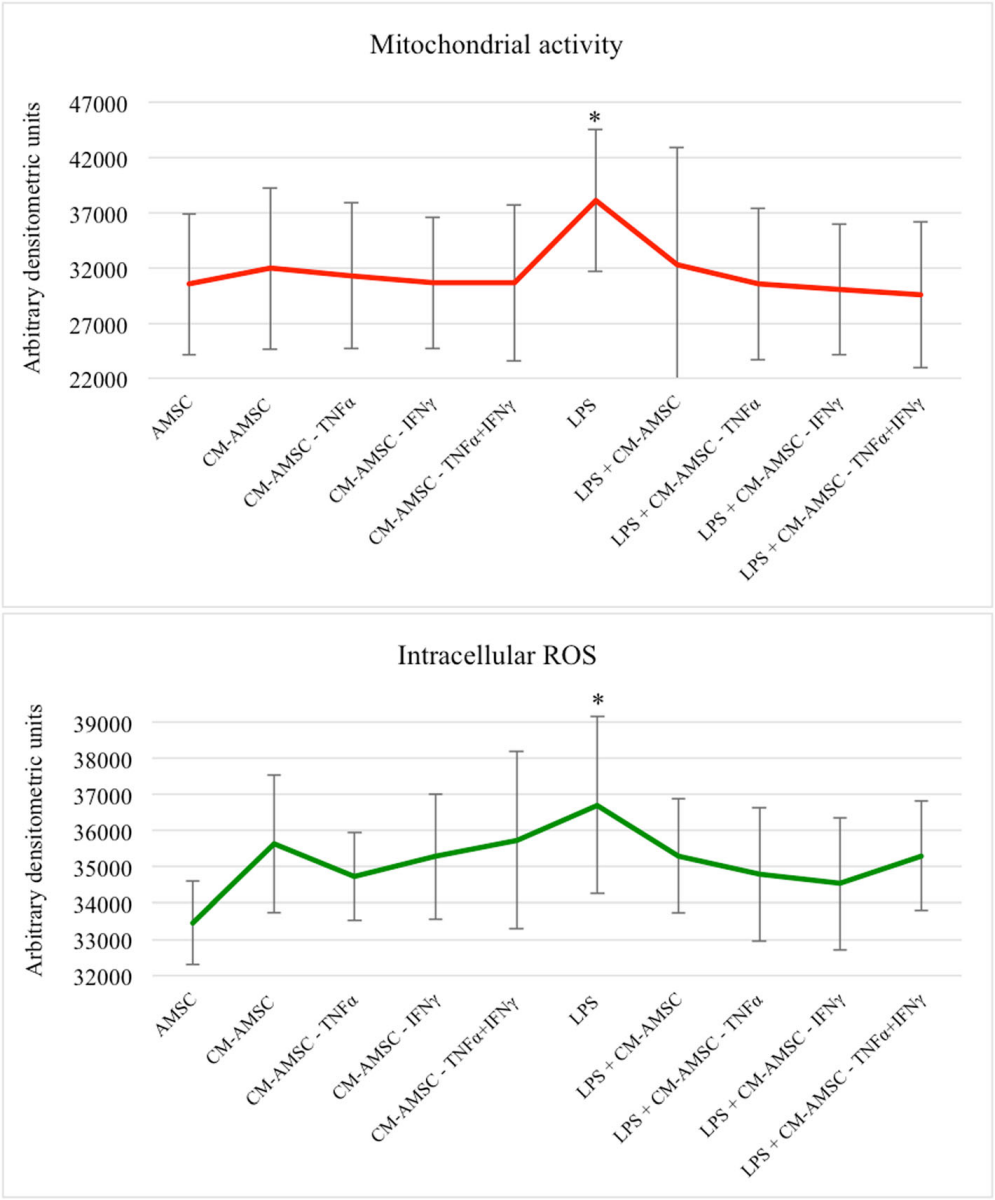
Mitochondrial activity and ROS levels are expressed as means ± SD of three different biological replicates with MitoTracker Orange (**a**) and dichlorodihydrofluorescein diacetate (DCDHFDA; **b** fluorescence intensity in arbitrary densitometric units (ADU). LPS-treated cells showed significantly increased mitochondrial and ROS levels compared with controls. The addition of CM alone or in combination with TNF and/or IFN restored the basal bioenergy status of endometrial cells. One-way ANOVA followed by Tukey post hoc test: * for *P* < 0.05

**Fig. 6:**
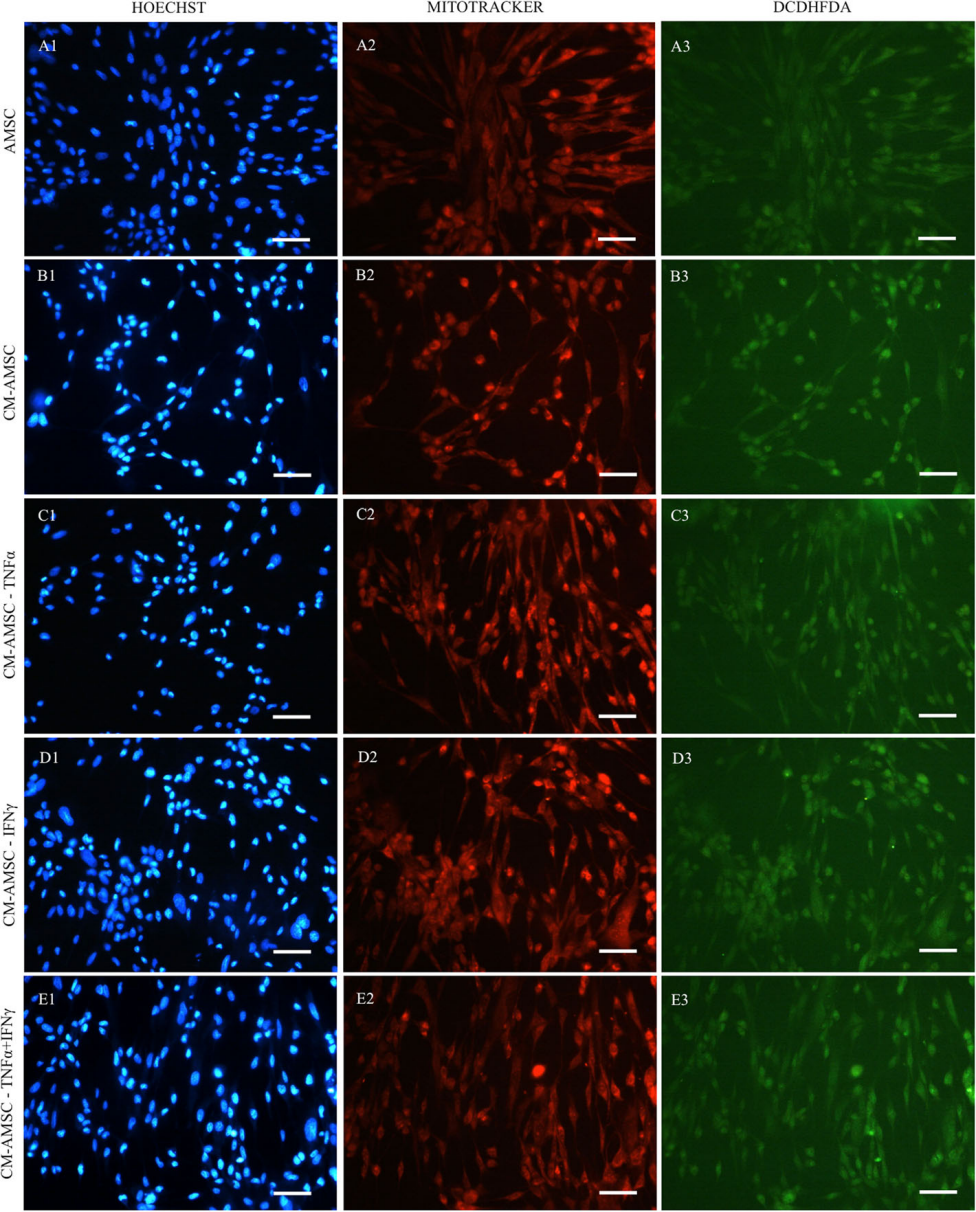
Photomicrographs representative of three different experimental conditions: AMSC (lane A), CM-AMSC (lane B), CM-TNF-α (lane C), CM-IFN-γ (lane D), and CM-TNF-α+IFN-γ (lane E). Corresponding epifluorescence images showing cell nuclei (column 1: Hoechst 3258) and epifluorescence images showing mitochondrial activity (column 2: MitoTracker Orange) and intracellular ROS levels (column 3: H2DCF-DA). Scale bars represent 20 μm

**Fig. 7:**
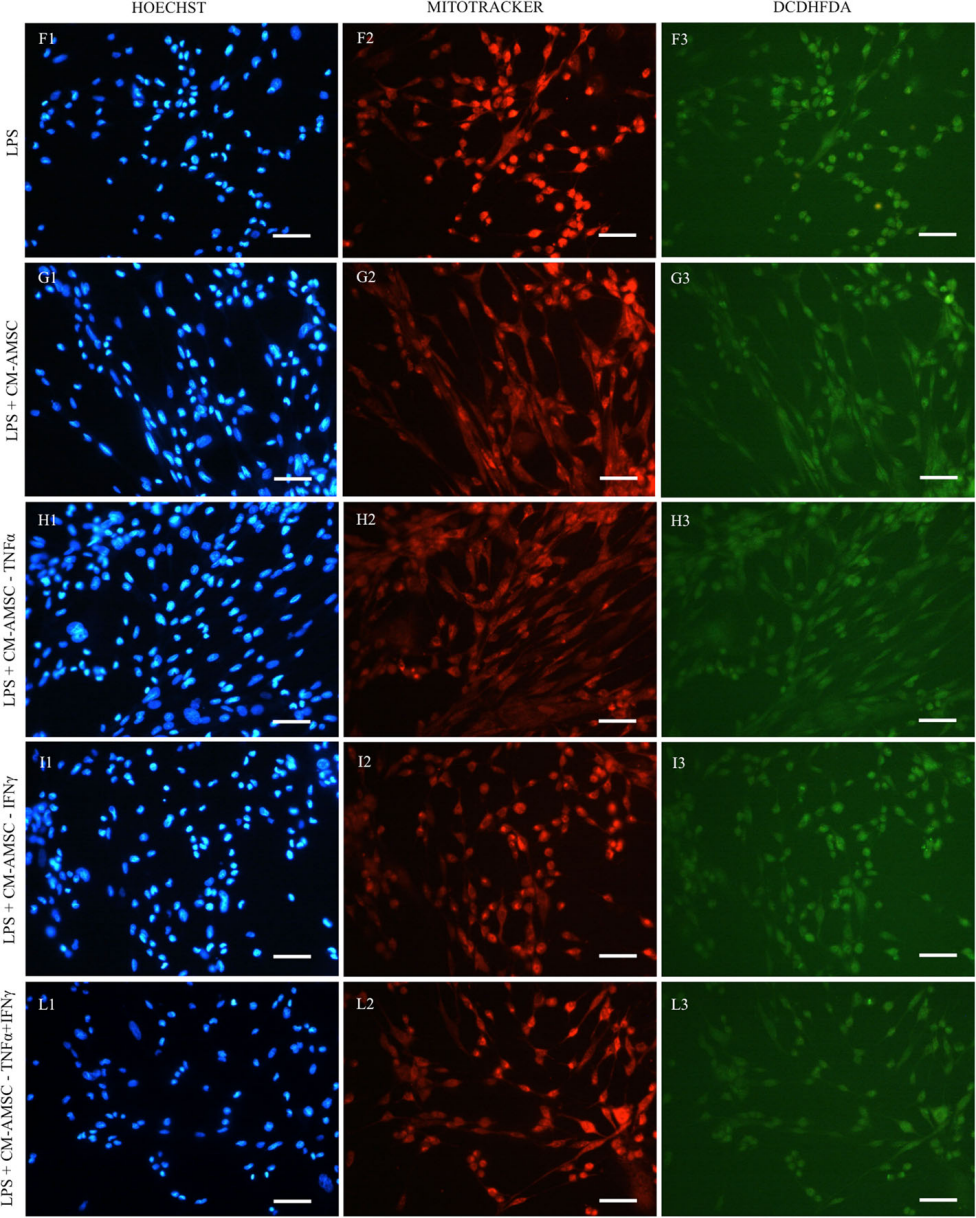
Photomicrographs representative of three different experimental conditions: LPS (lane A), LPS-CM-AMSC (lane B), LPS-CM-TNF-α (lane C), LPS-CM-IFN-γ (lane D), and LPS-CM-TNF-α+IFN-γ (lane E). Corresponding epifluorescence images showing cell nuclei (column 1: Hoechst 3258) and epifluorescence images showing mitochondrial activity (column 2: MitoTracker Orange) and intracellular ROS levels (column 3: H2DCF-DA). A slight increase of mitochondrial activity and intracellular ROS levels can be seen in the LPS-exposed cells (F2, F3) compared with the other conditions. Scale bars represent 20 μm

To establish the mechanisms underlying the damaging effects of LPS on endometrial cells, the energy/oxidative conditions of the cells exposed to different stimuli were investigated. We found that exposure of endometrial cells to LPS induced a substantial modification of the bioenergetic/oxidative status of this cell line. In fact, it increased both the mitochondrial activity (Fig. 5, panel a) and intracellular ROS levels (Fig. 5, panel b), compared with the basal, naïve AMSCs (CTR), and condition (*P* < 0.05). Moreover, it was interesting to find that LPS-stressed endometrial cells treated with CM derived from cells primed with TNF-α or IFN-γ or their combination restored their bioenergetic/oxidative status (by reducing mitochondrial activity and ROS levels) to values similar to those identified in the naïve AMSCs (CTR) and CM-AMSC (*P* < 0.05). Figures 6 and 7 show representative images of endometrial cells analyzed under an epifluorescence microscope. Increased fluorescence intensities of mitochondria- and ROS-specific probes are visible in the LPS-exposed sample compared with CTR, CM-AMSC, and LPS+CM conditions.

## Discussion

In MSC-based therapy, the priority is to develop in vitro culture methods that mimic the natural MSC niche, allowing cell expansion for clinical use, while maintaining cell quality and function. In recent years, priming approaches to increase the therapeutic potential of MSCs have been investigated. Priming (also referred to as licensing or preconditioning) with pro-inflammatory mediators consists of preparing cells for some specific function or lineage-specific differentiation, which involves cell activation, molecular signaling, genetic, or epigenetic modifications [16]. This strategy is effective in promoting immunoregulatory properties when compared to unprimed cells; indeed, adipose tissue-derived MSCs are not able to inhibit proliferation of PBMCs without exposure to IFN-γ indicating that inhibitory factors are released on MSC activation [34]. In addition, others have shown a significantly enhanced immune-suppressive potential for the umbilical cord- and bone marrow-derived MSCs after IFN-γ stimulation [35]. Specifically, several studies indicate that the human bone marrow-derived cells (BM-MSCs [18, 36–39] and equine BM-MSCs [15, 40]) need to be “licensed” by inflammatory signaling to become fully immunosuppressive.

Conversely, we have previously demonstrated that naïve equine AMSCs can inhibit the proliferation of equine PBMCs when cultured in both cell-to-cell contacts and when separated by a transwell membrane, suggesting that intrinsically secreted factors are involved in the process. Accordingly, we also demonstrated that the proliferation of PBMCs was also inhibited by the CM generated from naïve AMSC [19].

In the present paper, AMSC priming did not enhance their ability to inhibit lymphocyte proliferation compared to controls. The only statistically significant difference was with AMSCs primed with both TNF-α and IFN-γ. Our previous study reported that CM from horse AMSCs significantly reduced the production of TNF-α, together with a trend to reduction of TGF-β and IL-6 release from alveolar macrophages stimulated with LPS [41], and it remains to be investigated whether or not priming could enhance these effects.

Double primed AMSCs were able to inhibit PBMC proliferation, even if the AMSC viability was lower than the other experimental conditions (58%). It is known that inflammatory exposure can diminish MSC viability [42], even though the effect of priming on AMSC survival in vivo remains to be determined.

It is possible that there are other mechanisms by which MSCs participate in regeneration [43]; indeed, CM obtained from both naive and primed AMSCs was able to strongly inhibit PBMC proliferation, suggesting that the key mediators for the inhibition of proliferation are constitutively expressed not only in the AMSCs but also in their secretome. These data confirm our previous observations demonstrating that CM from human AMSCs inhibits the proliferation of PBMCs without priming [2].

Herein, we also studied whether priming makes the AMSCs more responsive, by evaluating the effect of priming on cell viability and oxidative stress using a model of LPS-induced injury in endometrial cells. In endometrial cells, LPS induces damage at different levels, leading to mitochondrial dysfunction and ROS over-production. We have shown that CM from naïve and primed AMSCs has a similar ability to increase the viability of LPS-stressed endometrial cells, thus suggesting that activation with pro-inflammatory cytokines is not a prerequisite to make the secretome more responsive to inflammatory environments. After LPS stimulation, only 21.66 ± 8.43% of endometrial cells survived while approximately 65% of cells survived after treatment with CM obtained with different activations.

These data were confirmed by evaluating the bioenergetic/redox status of the endometrial cells. Oxidative stress is one of the main pathophysiological mechanisms for some inflammatory conditions [44]. LPS can induce increased oxidative stress and mitochondrial dysfunction, characterized by a decrease in mitochondrial protein expression, mitochondrial mass, and reduction in the mitochondrial membrane potential [44]. Our data demonstrates that CM derived from amniotic cells has beneficial effects on the bioenergetic/oxidative status of LPS-stressed endometrial cells. In fact, while LPS led to excessively driven mitochondria activity with consequent ROS generation, CM restored the basal bioenergy status identified in the CTR condition and there was no statistical difference between CM from naïve and primed cells. This suggests the paracrine mechanism of AMSCs, as we previously demonstrated by CM administration in spontaneous tendon lesions in sport horses [19], and by culturing endometrial cells in vitro in the presence of CM, where a significant increase in proliferation rate occurred. Thus, there is the potential to use the CM in vivo to improve endometrial cell replenishment [44].

To date, many pro-inflammatory cytokines or growth factors are used for priming MSCs isolated from different species and/or tissues. Also, for MSCs from different equine tissues, pro-inflammatory priming increases immunosuppressive properties by stimulating the secretion of anti-inflammatory and immunomodulatory factors [45]. The fact that we demonstrate that AMSCs do not require priming could underline the different immunomodulatory profile of MSCs from different tissues. Interestingly, our observation that cells from the amniotic membrane possess intrinsic immunological properties could be explained by the role of the placenta in the feto-maternal tolerance process during the development and growth of a semi-allogeneic fetus during pregnancy. Indeed, a successful pregnancy is characterized by distinct immunological stages: the first is a pro-inflammatory phase required for implantation and placentation, this is followed by an anti-inflammatory phase allowing for fetal growth and development, and the final stage is a pro-inflammatory process that is required for parturition [46]. Therefore, it could be hypothesized that AMSCs are already intrinsically/naturally primed by the inflammatory placental microenvironment from which they derive.

This suggests that AMSCs may be immediately prone to downregulate the lymphocyte response to promote healing when placed in an inflammatory niche dominated by TNF-α and IFN-γ.

This could explain the difference in priming results between bone marrow-derived cells and AMSCs in equine species. The priming protocol used in our study was the same as that used by Hill et al. [18] to license equine bone marrow-derived cells. After a 96-h priming with IFN-γ, in bone marrow-derived cells, there was an increase in cell surface expression of MHCI and MHC II while TNF-α priming increased MHCI expression only. With the same concentration of cytokines, the AMSC phenotype was unchanged and is thus independent of the environment.

## Conclusions

In conclusion, the use of AMSCs may avoid the significant disadvantages associated with cell priming, such as the potential increase of immunogenicity [12, 15, 18], and the costs associated with cell culture and the use of expensive recombinant cytokines. The expression of MHCI and MHCII was similar to CTR after priming, confirming our previous cell characterization [21]. The use of AMSCs represents an alternative and promising source of MSCs and offers the potential for the production of cost-effective off-the-shelf cells and cell-free products from biological waste without priming.

## Data Availability

All data generated and/or analyzed during this study are included in this article.

## Abbreviations

ADU: Arbitrary densitometric units
AMSCs: Amniotic mesenchymal stromal cells
ANOVA: Analysis of variance
AO: Acridine orange
BM-MSCs: Bone marrow mesenchymal stromal cells
BSA: Bovine serum albumin
CCCP: Carbonyl cyanide 3-chlorophenyl hydrazone
CCL2: C-C motif chemokine ligand 2
cDNA: Complementary DNA
CM: Conditioned *medium*
CM-AMSC: CM derived from naïve AMSCs
CT: Threshold cycle
DCF: 2′,7′-Dichlorofluorescein
DMSO: Dimethyl sulfoxide
EDTA: Ethylenediaminetetraacetic acid
FBS: Fetal bovine serum
GAPDH: Glyceraldehyde 3-phosphate dehydrogenase
H2DCF: 2′,7′-Dichlorodihydrofluorescein
H2DCF-DA: 2′,7′-Dichlorodihydrofluorescein diacetate
HG-DMEM: High-glucose Dulbecco’s modified Eagle’s *medium*
HGF: Hepatocyte growth factor
HOXA-9: Homeobox protein hox-a9-like
IDO: Indoleamine 2,3-dioxygenase
IFN-γ: Interferon-γ
LPS: Lipopolysaccharide
MHC: Major histocompatibility complex
MPR: Membrane-associated progesterone receptor
MSC1: Mesenchymal stromal cells with pro-inflammatory phenotype
MSC2: Mesenchymal stromal cells with immunosuppressive phenotype
MSCs: Mesenchymal stromal cells
P/S: Penicillin/streptomycin
PBMCs: Peripheral blood mononuclear cells
PBS: Phosphate buffer solution
PGE2: Prostaglandin-E2
PGRMC1: Membrane-bound intracellular progesterone receptor component 1
PHA: Phytohemagglutinin
PI: Propidium iodide
PR: Progesterone receptor
qPCR: Real-time quantitative polymerase chain reaction
RNA: Ribonucleic acid
ROS: Reactive oxygen species
RT-PCR: Reverse transcription-polymerase chain reaction
TGF-β1: Transforming growth factor β1
TLR: Toll-like receptor
TNF-α: Tumor necrosis factor-α
